# Prognostic implications of CD123 in pediatric B-cell acute lymphoblastic leukemia: a single-center retrospective analysis

**DOI:** 10.3389/fped.2026.1887012

**Published:** 2026-08-12

**Authors:** Zheng Li, Qinfa Chen, Zhiyong Zhou, Fei He

**Affiliations:** Department of Hematology, Jiangxi Provincial Children’s Hospital, Nanchang, China

**Keywords:** B-cell acute lymphoblastic leukemia, CD123, children’s oncology group, pediatric, prognostic

## Abstract

**Introduction:**

Acute lymphoblastic leukemia (ALL) is the most common pediatric hematologic malignancy, with the majority of cases being of B-cell origin. While many patients respond favorably to therapy, a subset experiences early relapse or poor outcomes, underscoring the critical need for reliable prognostic biomarkers. This study investigated the significance of CD123 expression in diagnosing the disease, evaluating therapeutic efficacy, and predicting prognosis in pediatric B-cell ALL (B-ALL).

**Methods:**

This retrospective study included 305 pediatric patients with B-ALL admitted to our hematology department from January 2016 to December 2021. All patients were treated following the CCCG-ALL-2015 protocol. Based on flow cytometric evaluation, patients with ≥20% or <20% CD123-expressing blasts were stratified into CD123-positive (CD123^+^) and CD123-negative (CD123^−^) groups, respectively.

**Results:**

CD123 was expressed on the leukemic blasts of 60.3% of patients, exhibiting a strong positive correlation with CD34 expression (*P* < 0.001). CD123 positivity was substantially associated with specific genetic abnormalities. Specifically, the frequencies of the *ETV6::RUNX1* and *TCF3::PBX1* fusion genes were significantly lower in the CD123^+^ group than in the CD123^−^ group. The 5-year event-free survival (EFS) rate was significantly higher in the CD123^+^ group than in the CD123^−^ group (*P* = 0.027). This favorable prognostic impact was particularly pronounced in patients exhibiting minimal residual disease (MRD) positivity on day 19 (*P* = 0.006). Furthermore, combining CD123 status with day 19 MRD enabled more accurate identification of patients with a poor prognosis.

**Discussion:**

CD123 positivity on leukemic blasts correlates with distinct genetic profiles and favorable clinical outcomes in pediatric B-ALL. Ultimately, integrating CD123 status with early treatment response facilitates accurate risk stratification and guides personalized therapeutic strategies.

## 1 Introduction

Acute lymphoblastic leukemia (ALL) represents the predominant form of childhood cancer, constituting 25% of all pediatric malignancies diagnosed before the age of 15 (1). Among these cases, B-cell ALL (B-ALL) makes up roughly 85% (2). In most developed nations, the overall cure rate for ALL currently exceeds 90%. This favorable outcome is largely due to precise risk-adapted patient stratification and the integration of prognostic indicators—including age, white blood cell (WBC) count, cytogenetic abnormalities, and minimal residual disease (MRD)—into personalized treatment regimens (3, 4). Nevertheless, treatment responses can differ significantly even among patients within the same risk category; notably, a certain proportion of low-risk individuals fail to attain bone marrow remission or suffer from early relapse (5, 6). Such clinical heterogeneity underscores the necessity of improving current risk stratification systems through the discovery of novel prognostic biomarkers.

CD123, also known as the alpha subunit of the interleukin-3 receptor (IL-3Rα), is upregulated across a range of malignant cell types and has been implicated in the pathogenesis of both myeloid and lymphoid neoplasms (7). In patients with ALL or acute myeloid leukemia (AML), CD123 is present on the majority of leukemic blasts, making it a useful marker for MRD evaluation (8). Notably, while expressed on normal hematopoietic stem cells, CD123 is not overexpressed on these cells, a feature that sets them apart from leukemic stem cells (LSCs) from a biological perspective (9). As a result, CD123 holds promise as a biomarker for cancer diagnosis, outcome prediction, and therapeutic monitoring (10, 11). To date, research has predominantly examined CD123 in the context of AML. In this disease, elevated CD123 levels correlate with heightened resistance to chemotherapy and the presence of high-risk genetic lesions, both of which are strongly linked to unfavorable clinical outcomes (12, 13). Recently, CD123-directed therapies—including specific antibodies and chimeric antigen receptor (CAR) T cells targeting this antigen—have been explored for AML management (14–16). Although CD123 is similarly expressed on ALL blasts, its prognostic significance in pediatric ALL remains poorly understood. A deeper understanding of how CD123 contributes to the onset and persistence of hematologic cancers could offer critical clinical insights and open new therapeutic avenues for patients with substantial unmet treatment needs. In this retrospective study, we analyzed CD123 expression and its correlation with clinical characteristics and prognosis in 305 patients with newly diagnosed B-ALL admitted to Jiangxi Children's Hospital from January 2016 to December 2021.

## 2 Materials and methods

### 2.1 Patients

This retrospective study enrolled 305 patients with newly diagnosed B-ALL who were admitted to Jiangxi Provincial Children's Hospital between January 2016 and December 2021. Diagnoses were established following the 2008/2017 WHO Classification of Tumors of Hematopoietic and Lymphoid Tissues, based on standard assessments of cell morphology, cytogenetics, immunophenotyping, and molecular testing. All patients received treatment according to the CCCG-ALL-2015 regimen. Comprehensive details regarding the inclusion and exclusion criteria, diagnostic workup, risk assignment, treatment protocols, and disease surveillance have been previously reported (17). Written informed consent was obtained from the patients' legal guardians, and the study protocol received approval from the Medical Ethics Committee of Jiangxi Provincial Children's Hospital (approval no. JXSETYY-YXKY-20250087).

### 2.2 Assessment of CD123 expression

At the time of initial diagnosis and prior to induction chemotherapy, 2–3 mL of heparinized bone marrow specimens were obtained. After cell counting, the concentration was adjusted to 10–20 × 10^9^cells/L, followed by a standard stain-lyse-wash protocol. In each analysis tube, 50 µL (equivalent to 1 × 10^6^ cells) was stained with a panel of monoclonal antibodies, including CD45-FITC, CD10-PE, CD34-PerCP, and CD19-APC. Additional markers conjugated to FITC or phycoerythrin were also assessed, such as CD123, TdT, cyIgM, sIgM, CD20, cyCD22, CD22, and cyCD79a—all of which are frequently used MRD-related markers in B-ALL. The anti-CD123 antibody was obtained from BD Biosciences [340545; Clone 9F5 (RUO, GMP); San Diego, CA, USA], and a mouse BALB/c IgG1 isotype antibody served as the negative control [550617; Clone MOPC-31C (RUO); BD Biosciences]. Blast populations were identified via CD45 vs. side scatter gating. CD123 expression levels on the gated blasts were evaluated using a Beckman Coulter flow cytometer (Beckman Coulter, USA), with data analysis performed using BD FACSDiva™ software (BD Biosciences, CA, USA). A sample was defined as CD123-positive when ≥20% of the blast population expressed this marker (18).

### 2.3 Assessment of treatment response and outcomes

Treatment efficacy was assessed in accordance with the CCCG-ALL-2015 protocol, using MRD monitoring at two time points: day 19 (D19) and day 46 (D46) of induction chemotherapy. All enrolled patients were required to undergo MRD evaluation on D19. Additionally, MRD assessment on D46 was mandated for patients belonging to the intermediate- or high-risk (IR/HR) categories, as well as for those in the low-risk (LR) group who exhibited a D19 MRD level of 0.1% or higher. Overall survival (OS) was defined as the interval from the date of diagnosis to either death or the last follow-up. Event-free survival (EFS) was measured from diagnosis until the first occurrence of any of the following events: disease relapse, refractory disease, death from any cause, secondary malignancy, or treatment cessation due to severe toxicity. Patients who did not experience any such events were censored at the time of their last follow-up. Those who discontinued treatment for reasons other than severe toxicity, were lost to follow-up, or were transferred to another institution were censored on the date of their last known contact. The cutoff date for follow-up data collection was January 2024; follow-ups were conducted via telephone interviews and outpatient clinic visits.

### 2.4 Statistical analysis

Quantitative data are presented as mean ± standard deviation, median, count, or percentage, as appropriate. Comparisons of categorical variables were conducted using the chi-square test. Survival probabilities were estimated via the Kaplan–Meier method, and differences in survival rates between groups were assessed using the log-rank test. Multivariable analysis was carried out using Cox proportional hazards regression. A two-tailed *P* value of less than 0.05 was considered statistically significant. All statistical analyses were performed with SPSS Statistics version 22.0 (IBM Corp., Armonk, NY, USA). Graphical illustrations were created using GraphPad Prism 8.3.0 (GraphPad Software, San Diego, CA, USA).

## 3 Results

### 3.1 Study population

Among the 305 patients with B-ALL, 184 (60.3%) were CD123-positive (CD123^+^) and 121 (39.7%) were CD123-negative (CD123^−^) (Table 1). Clinical and biological characteristics were compared between the CD123^+^ and CD123^−^ groups (Table 2). The rate of CD34 positivity was significantly higher in the CD123^+^ group than in the CD123^−^ group (85.3% vs. 57.9%, *P* < 0.001). Conversely, the frequencies of the *ETV6::RUNX1* (14.7% vs. 29.8%, *P* = 0.002) and *TCF3::PBX1* (0.5% vs. 16.5%, *P* < 0.001) fusion genes were significantly lower in the CD123^+^ group. No significant differences were observed between the cohorts regarding other clinical characteristics, such as sex, age at diagnosis, initial WBC count, or risk stratification (*P* > 0.05).

**Table 1:** Clinical characteristics of patients in the study population.

Characteristics	ALL patients (*N* = 305)	Characteristics	ALL patients (*N* = 305)
Age (y), median (IQR)	4.5 (3.2–7.4)	CD34, *n* (%)	
<10, *n* (%)	267 (87.5)	Positive*	227 (74.4)
≥10, *n* (%)	38 (12.5)	Negative	75 (24.6)
Gender, *n* (%)		Unknown	3 (1)
Female	131 (43.0)	Risk group, *n* (%)	
Male	174 (57.0)	Low	188 (61.6)
WBC (×10^9^/L), median (IQR)	8.8 (3.7–28.8)	Intermediate	115 (37.7)
<50, *n* (%)	259 (84.9)	High	2 (0.7)
≥50, *n* (%)	46 (15.1)	D19 MRD, *n* (%)	
Hb (g/L), median (IQR)	71 (55–89)	<0.01% (negative)	167
PLT (×10^12^/L), median (IQR)	45 (22–91)	≥0.01% (positive)	134
Genetic abnormality, *n* (%)		Unknown	4
*BCR::ABL1*	13 (4.3)	D46 MRD, *n* (%)	
*ETV6::RUNX1*	66 (21.6)	<0.01% (negative)	184
*TCF3::PBX1*	22 (7.2)	≥0.01% (positive)	33
*MLL-*rearranged	4 (1.3)	Unknown^#^	88
CD123, *n* (%)			
Positive	184 (60.3)		
Negative	121 (39.7)		

* Positive expression was considered cell antigen CD34 expression ≥20%.

# According to the CCCG-2015ALL protocol, only cases in the IR/HR group and the LR group with D19 MDR ≥ 0.1% need to undergo D46 MRD assessment.

**Table 2:** Correlation of CD123 expression with clinicopathological features of the patients.

Feature	CD123^+^ (*n* = 184)		CD123^−^ (*n* = 121)		*P*
	*N*	%	*N*	%	
Sex					
Male	103	56.0	71	58.7	0.641
Female	81	44.0	50	41.3	
Age, years					
<10	163	88.6	104	86.0	0.595
≥10	21	11.4	17	14.0	
WBC (×10^9^/L)					
<50	155	84.2	104	86.0	0.745
≥50	29	15.8	17	14.0	
Hb (g/L)					
Median (IQR)	67 (54, 87)		73 (53, 94)		0.162
PLT (×10^12^/L)					
Median (IQR)	45 (23, 93)		49 (21, 90)		0.756
BM,%					
Median (IQR)	83.0 (68.0, 88.0)		83.0 (68.8, 88.6)		0.962
CD34 positive	157	85.3	70	57.9	<0.001
Cytogenetics					
*BCR::ABL1*	6	3.3	7	5.8	0.386
*ETV6::RUNX1*	27	14.7	36	29.8	0.002
*TCF3::PBX1*	1	0.5	20	16.5	<0.001
KMT2A-rearranged	3	1.6	1	0.8	0.480
Risk group					
LR	113	61.4	75	62.0	0.920
IR/HR	71	38.6	46	38.0	
D19 MRD					
<0.01%	93	50.8	74	62.7	0.043
≥0.01%	90	49.2	44	37.3	
D46 MRD					
<0.01% (negative)	110	82.1	74	89.2	0.178
≥0.01% (positive)	24	17.9	9	10.8	

### 3.2 CD123 expression and clinical outcomes

**Figure 1:**
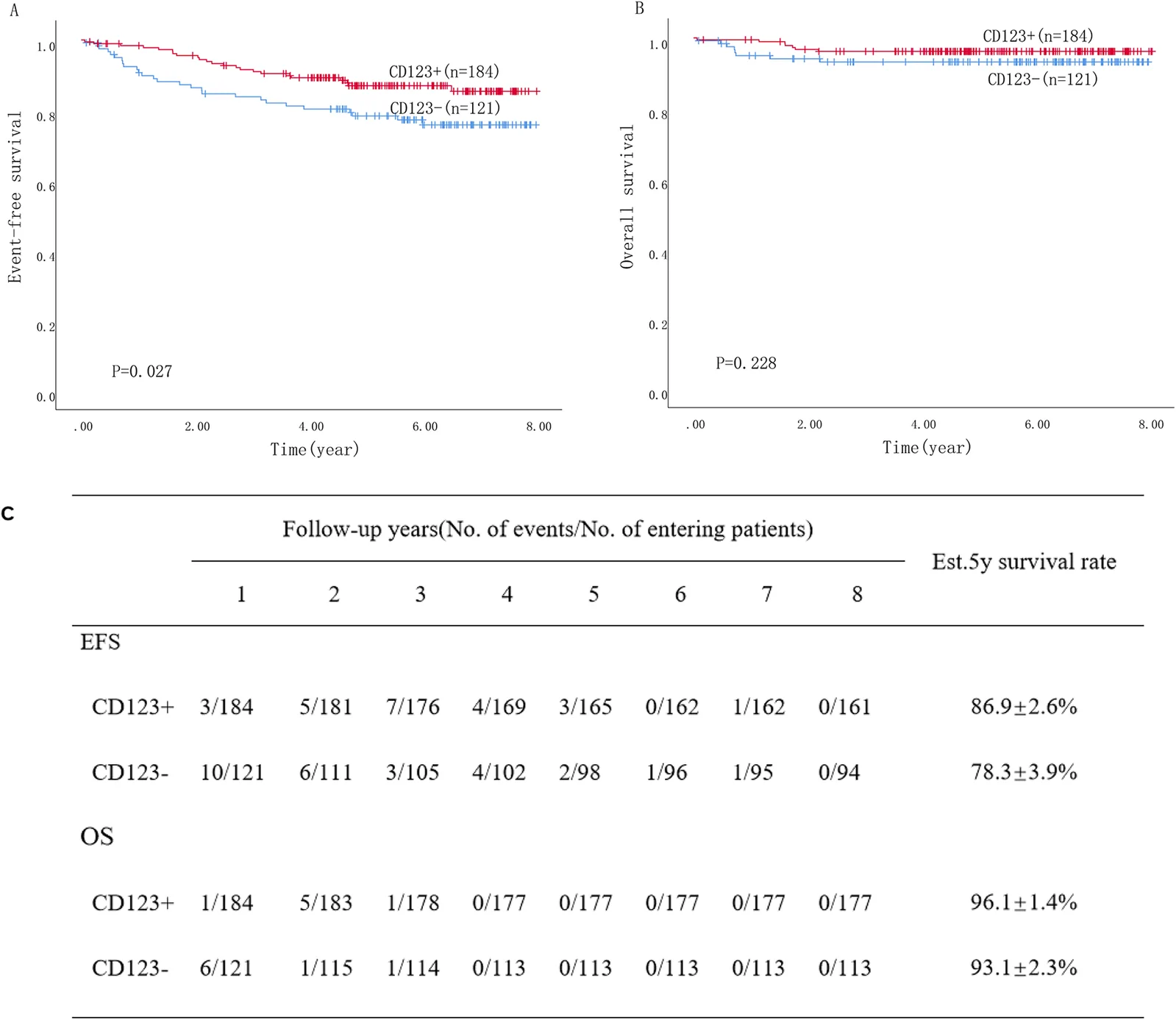
CD123 expression correlates with favorable clinical outcomes of pediatric B-ALL. Comparison of EFS **(A)** and OS **(B)** among CD123^+^ and CD123^−^ groups. **(C)** The detailed number of event occurrences and cases under observation in the CD123^+^ and CD123^−^ groups in every follow-up year. Estimated 5-year survival rates in each group were calculated.

We next evaluated the prognostic impact of CD123 expression on EFS and OS. The median follow-up duration was 54 months (interquartile range, 38.4–88.8; range, 0.7–97.3). Notably, 179 patients were followed for ≥5 years. The 5-year EFS rate was significantly higher in the CD123^+^ group than in the CD123^−^ group (89.6% ± 2.6% vs. 78.3% ± 3.9%, *P* = 0.027); however, no significant difference in OS was observed between the cohorts (Figure 1). We further integrated clinical and biological parameters to evaluate the prognostic impact of CD123 across specific patient subgroups (Table 3). The favorable prognostic impact of CD123 positivity was evident in patients diagnosed at age ≥10 years, those in the IR/HR groups, and those exhibiting D19 MRD positivity, regardless of genomic abnormalities. Univariable Cox regression analysis showed that CD123 positivity was a protective factor against adverse events. Other significant prognostic factors identified included age, D19 MRD, initial WBC count, risk stratification and genetic abnormalities (*ETV6::RUNX1* and *BCR::ABL1*). Multivariable analysis confirmed that CD123 positivity served as an independent protective factor against adverse events (Table 4).

**Table 3:** Precise prognostic features of CD123 in B-ALL.

Feature	*N* (CD123^+^/CD123^−^)	EFS, (95% CI)		*P*
		CD123^+^	CD123^−^	
Sex				
Male	103/71	83.5 (78.6–88.4)	78.0 (70.8–85.2)	0.090
Female	81/50	88.5 (83.5–93.6)	80.4 (72.4–88.4)	0.106
Age (year)				
＜10	163/104	86.7 (83.3–90.1)	81.4 (76.6–86.2)	0.432
≥10	21/17	88.4 (80.3–94.2)	64.9 (49.5–80.4)	0.001
WBC (×10^9^/L)				
＜50	155/104	83.8 (77.9–89.6)	76.1 (68.9–83.3)	0.056
≥50	29/17	87.4 (83.2–91.7)	83.6 (75.7–91.4)	0.139
Subtypes				
*BCR::ABL1*	6/7	50.4 (38.7–62.1)	52.7 (23.4–82.1)	0.163
*ETV6::RUNX1*	27/36	90.1 (86.4–93.8)	90.6 (85.2–96.0)	0.891
*TCF3::PBX1*	1/20	12.3 (12.3–12.3)	83.1 (69.1–97.1)	0.749
Risk group				
LR	113/75	90.7 (87.4–93.9)	86.0 (81.0–91.1)	0.354
IR/HR	71/46	78.9 (73.1–84.8)	72.7 (65.0–80.4)	0.014
Day 19 MRD				
＜0.01%	93/74	91.8 (88.2–95.3)	86.5 (80.9–92.2)	0.070
≥0.01%	90/44	78.8 (72.9–84.6)	66.8 (57.3–76.4)	0.006
Day 46 MRD				
＜0.01%	110/74	87.8 (83.6–92.0)	80.3 (73.3–87.3)	0.054
≥0.01%	24/9	79.9 (71.2–88.5)	71.7(73.3–87.3)	0.114

**Table 4:** Cox's proportional hazards regression model analysis of factor affecting event-free survival (EFS).

Variate	Univariate Cox's regression			Mulvariate Cox's regression		
	HR	95% CI	*P*	HR	95% CI	*P*
CD123 positive	0.54	0.31–0.95	0.029	0.39	0.11–0.73	0.003
Age ≥10 years	2.10	1.05–4.20	0.035	2.70	1.17–6.21	0.020
D19 MRD ≥0.01%	4.32	2.30–8.13	<0.001	7.55	2.80–20.35	<0.001
WBC ≥50 ×10^9^/L	1.92	1.00–3.67	0.048	1.27	0.56–2.86	0.572
D46 MRD ≥0.01%	2.92	1.48–5.74	0.002	1.49	0.71–3.13	0.289
IR/HR risk group	2.82	1.61–4.94	<0.001	1.34	0.64–2.86	0.441

### 3.3 New risk stratification

**Figure 2:**
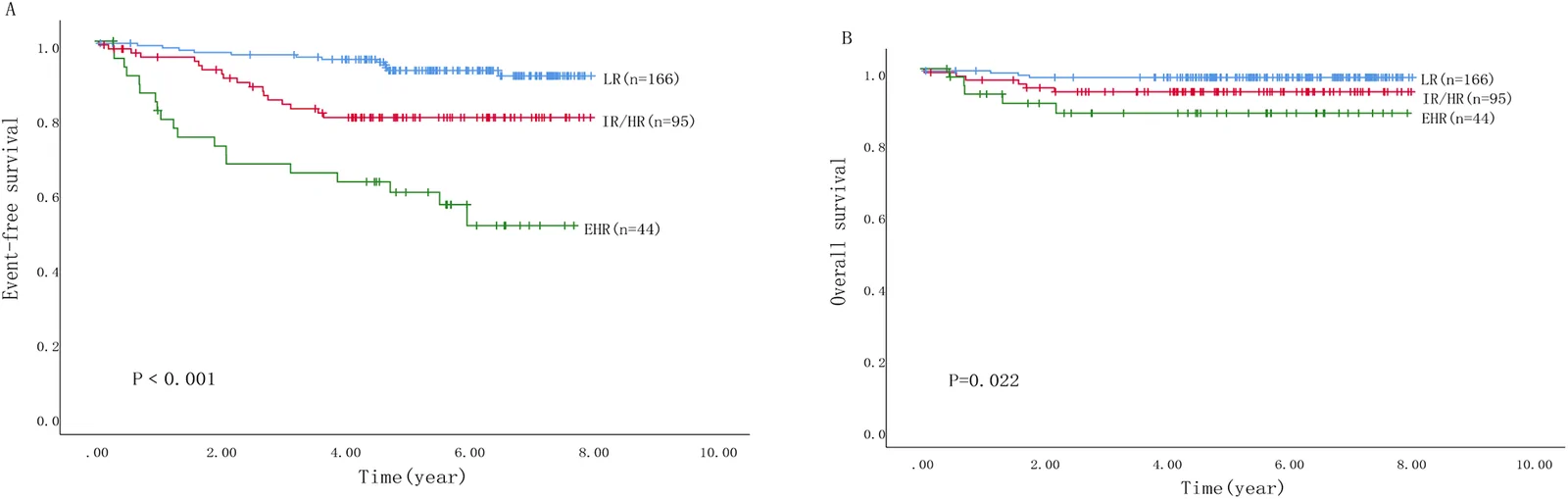
The impact of the new risk stratification on the EFS **(A)** and OS **(B)**.

Based solely on CD123 expression levels and D19 MRD status, we established an “extreme high risk” (EHR) group, defined exclusively by the combination of CD123 negativity and D19 MRD positivity (≥0.01%), independent of cytogenetic or molecular genetic markers. This group was compared with the conventional LR and IR/HR groups stratified by established genetic risk criteria (Table 5). The 5-year EFS rate was significantly lower in the EHR group (59.4% ± 7.6%) than in the LR (91.7% ± 2.2%) and IR/HR (79.9% ± 4.2%) groups (*P* < 0.001). Similarly, the 5-year OS rate in the EHR group (87.6% ± 5.2%) was markedly lower than those in the LR (97.6% ± 1.2%) and IR/HR (93.7% ± 2.5%) groups (*P* = 0.022) (Figure 2). These findings indicate that this combined risk stratification system effectively predicts clinical outcomes in pediatric patients with B-ALL.

**Table 5:** Risk stratification criteria for patient groups.

Stratification	Criteria
LR	Necessary condition (B-ALL meets one of the following conditions): ① Age≥365 days, ≤9.9 years old, and WBC ≤50 × 10^9^/L; ② Chromosome number ≥50 or DNA index ≥1.16; ③ TEL-AML1; The following situations must be excluded: ① CNS3 and/or testicular leukemia ② *t*(1;19), *t*(9;22), MLLr, Chromosome number <44, iAMP21
IR	Ph^＋^ ALL; T-ALL; MLLr: age ≥6 months or WBC <300 × 10^9^/L; Chromosome number <44; All other cases of ALL that do not fall into the low-risk or high-risk categories
HR	D46 MRD ≥1%; MLLr-ALL: age <6 mouths, and WBC ≥300 × 10^9^/L
EHR	The blast population expressed CD123 <20% and D19 MRD ≥0.01%

## 4 Discussion

In this study, we report the flow cytometric quantification of surface CD123 expression on leukemic blasts from pediatric patients with newly diagnosed B-ALL treated under the CCCG-ALL-2015 protocol. We observed that 60.3% of the cohort exhibited CD123^+^ (≥20%) bone marrow blasts. CD123 expression correlated significantly with CD34 positivity and D19 MRD levels. No significant associations were found between CD123 expression and presenting age, sex, initial WBC count, risk group, or D46 MRD status. Furthermore, CD123 expression was closely associated with specific genetic variation profiles in pediatric B-ALL. Specifically, the frequencies of the *ETV6::RUNX1* and *TCF3::PBX1* fusion genes were significantly lower in the CD123^+^ group than in the CD123^−^ group. CD123 expression is closely linked to genetic alterations in leukemic blasts. For example, among 1,040 pediatric patients with AML, high CD123 expression correlated with a higher incidence of KMT2A rearrangements and FLT3-ITD mutations (12). Similarly, Angelova et al. reported that in adult patients with ALL, CD123 expression was more common in *BCR::ABL1*^+^ B-ALL than in *BCR::ABL1*^−^ B-ALL (19). Using multiparameter immunophenotyping, Wang et al. found that CD123 expression was significantly higher in patients with ZNF384 rearrangements than in those without the fusion (20).

CD123, also designated as the interleukin-3 receptor alpha chain (IL-3Rα), is a 75-kDa glycoprotein whose encoding gene resides within the pseudoautosomal regions of chromosomes Xp22.3 and Yp11.3. Upon forming a heterodimeric complex with the common β subunit (βc), CD123 enables high-affinity binding of IL-3 and stabilizes the IL-3/IL-3R complex. This assembly subsequently triggers downstream signaling cascades, including the Janus kinase/signal transducer and activator of transcription (JAK/STAT), mitogen-activated protein kinase (MAPK), and phosphoinositide 3-kinase/protein kinase B (PI3K/AKT) pathways (21, 22). These signaling events are essential for regulating hematopoietic development and facilitating the terminal differentiation of myeloid lineage cells (23, 24).

Because genomic abnormalities drive oncogenesis, specific cytogenetic profiles are critical for risk stratification due to their unique prognostic implications. In this study, the 5-year EFS rate of the CD123^+^ group was significantly higher than that of the CD123^−^ group. Both univariate and multivariate regression analyses validated that CD123 positivity independently predicts favorable prognosis in pediatric B-ALL. This beneficial effect was especially pronounced among patients aged 10 years or older, those classified into intermediate- or high-risk strata, and those with positive D19 MRD status. We acknowledge that several subgroup analyses presented in this study involved relatively small sample sizes, which may limit the statistical power to detect meaningful differences or to draw definitive conclusions. Therefore, these subgroup findings should be considered exploratory in nature and hypothesis-generating rather than confirmatory. Larger multicenter prospective cohorts are warranted to validate our observations in specific patient subsets. Conversely, a retrospective study involving 1,040 pediatric patients with AML identified CD123 as a marker of relapsed or refractory disease (12). Nevertheless, a retrospective study involving 976 pediatric B-ALL cases revealed that elevated CD123 levels on leukemia blasts correlated with better clinical prognoses (18). The IL-3/CD123 signaling pathways appear to function differently in myeloid vs. lymphoid lineages. In AML blasts, IL-3 stimulation induces strong activation of STAT5—a key transcription factor in the IL-3 pathway—indicating that IL-3Rα is crucial for the IL-3-mediated proliferation of these cells (25, 26). Conversely, in leukemic B cells, both the high expression of CD123 and the strong binding affinity of IL-3 for its receptors (CD123 and CD131) dictate cellular responses. In B lymphocytes that respond to IL-3, their generation is restricted to cells exhibiting low or negligible levels of IL-3Rα. For leukemic B-cell precursors sensitive to IL-3, binding to the low-affinity receptor (CD123) alone is adequate to drive IL-3-mediated proliferation (27).

Importantly, the prognostic significance of CD123 was profoundly amplified when combined with D19 MRD status. Under the CCCG-ALL-2015 protocol, sequential MRD monitoring on D19 and D46 dictates timely adjustments to risk stratification, ensuring precise risk assignment and optimized therapy (3). In our cohort, 44 patients meeting both phenotypic criteria—D19 MRD positivity and CD123 negativity—were classified into the EHR group. Notably, this classification was entirely based on flow cytometric parameters rather than genetic aberrations, yet this group experienced markedly inferior survival outcomes. Crucially, under the conventional protocol, more than half of these patients were assigned to the LR group. Incorporating CD123 status into existing frameworks resolves this critical misclassification, enabling clinicians to identify high-risk pediatric patients earlier and with greater accuracy.

Paradoxically, the D19 MRD positivity rate was significantly higher in the CD123^+^ group than the CD123^−^ group, indicating an initially poor response to induction chemotherapy. However, this discrepancy resolved by D46; thereafter, the treatment responses of the CD123^+^ cohort ultimately surpassed those of the CD123^−^ cohort. We hypothesize that high CD123 expression may confer transient chemoresistance during induction, followed by heightened chemosensitivity during the consolidation and maintenance phases. Consistent with this observation, elevated CD123 levels on AML leukemic stem cells result in high expression across the bulk tumor cell population, reflecting an arrested state at an early stage of differentiation that is associated with increased resistance to chemotherapy (28, 29). A comparable mechanism may similarly influence chemosensitivity in pediatric ALL.

Several limitations of this study warrant consideration. First, its retrospective, single-center design inherently introduces potential selection bias and limits the generalizability of our findings to broader populations or alternative treatment regimens. Second, and most critically, the proposed “Extreme High Risk” (EHR) classification—combining CD123 status and D19 MRD—was both derived and internally validated within the same cohort. Consequently, although our results are encouraging, the clinical applicability of this stratification model should be viewed with caution, and independent external validation in prospective, multicenter settings is indispensable prior to its adoption in routine clinical practice. Third, the dynamic alterations of CD123 expression during disease evolution, as well as the epigenetic mechanisms governing its regulation, remain poorly understood and merit further exploration. Moreover, the intratumoral heterogeneity of CD123 expression at the single-cell level—potentially resolvable by advanced single-cell sequencing technologies—adds another layer of complexity that deserves dedicated investigation. Finally, establishing standardized detection protocols and unified cut-off thresholds will be essential to facilitate clinical trials evaluating CD123-targeted therapies—particularly dual-target CAR-T cells—in pediatric ALL.

In conclusion, CD123 expression positivity functions as an independent prognostic indicator associated with favorable outcomes in pediatric B-ALL. Integrating CD123 status with D19 MRD monitoring accurately identifies high-risk patients who would otherwise be misclassified by conventional parameters. Given its clinical accessibility, CD123 expression profiling deserves further prospective evaluation as a potential adjunct to existing risk stratification algorithms.

## Data Availability

The datasets presented in this study can be found in online repositories. The names of the repository/repositories and accession number(s) can be found in the article/Supplementary Material.
